# Integrated Analysis to Obtain Potential Prognostic Signature in Glioblastoma

**DOI:** 10.3389/fnint.2021.717629

**Published:** 2022-01-05

**Authors:** Jia-Qi Chen, Nuo Zhang, Zhi-Lin Su, Hui-Guo Qiu, Xin-Guo Zhuang, Zhi-hua Tao

**Affiliations:** ^1^Department of Laboratory Medicine, The Second Affiliated Hospital of Zhejiang University, Hangzhou, China; ^2^Department of Clinical Laboratory, The People’s Hospital of Lishui, Lishui, China; ^3^Beijing Rehabilitation Hospital of Capital Medical University, Beijing, China; ^4^Department of Laboratory Medicine, The First Affiliated Hospital of Xiamen University, Xiamen, China

**Keywords:** glioblastoma multiforme, microenvironment, WGCNA, prognostic biomarkers, estimate

## Abstract

Glioblastoma multiforme (GBM) is the most malignant and multiple tumors of the central nervous system. The survival rate for GBM patients is less than 15 months. We aimed to uncover the potential mechanism of GBM in tumor microenvironment and provide several candidate biomarkers for GBM prognosis. In this study, ESTIMATE analysis was used to divide the GBM patients into high and low immune or stromal score groups. Microenvironment associated genes were filtered through differential analysis. Weighted gene co-expression network analysis (WGCNA) was performed to correlate the genes and clinical traits. The candidate genes’ functions were annotated by enrichment analyses. The potential prognostic biomarkers were assessed by survival analysis. We obtained 81 immune associated differentially expressed genes (DEGs) for subsequent WGCNA analysis. Ten out of these DEGs were significantly associated with targeted molecular therapy of GBM patients. Three genes (S100A4, FCGR2B, and BIRC3) out of these genes were associated with overall survival and the independent test set testified the result. Here, we obtained three crucial genes that had good prognostic efficacy of GBM and may help to improve the prognostic prediction of GBM.

## Introduction

Glioblastoma multiforme (GBM) is the most malignant and multiple tumors of the central nervous system (CNS), which is classified as grade IV glioma by the World Health Organization (WHO) (Ostrom et al., 2013; Louis et al., 2016; Hanif et al., 2017). GBM is a heterogeneous disease involving multiple subtypes with different clinical and molecular characteristics (Friedmann-Morvinski, 2014; Lee et al., 2018). The diagnosis of GBM is based on grading and histomorphology. However, the classification does not predict clinical outcomes after GBM development (Sasmita et al., 2018). To date, there was almost no biomarker that could translate into a significant survival benefit to GBM patients and the median survival of patients was only 15 months (Zhao et al., 2019).

In GBM, tumor cells interact with resident cells (neurons, glial cells, etc.) entangled in the extracellular matrix (ECM) and vascular system (De Luca et al., 2018). Glial cells play an important role in cancer progression (Friedmann-Morvinski et al., 2012). The peritumor tissue microenvironment is key to current and future research on tumor-sensitive therapies. GBM can affect the cellular morphology and function of the CNS through intercellular interactions (Martinez-Outschoorn et al., 2014). Glial cells are inextricably linked to the GBM, and their immune role has been well documented. Microglia and macrophages can rapidly respond to alterations in CNS homeostasis, including brain tumors. Microglia and macrophages have also been found to induce GBM cell cycle arrest and differentiation (Sarkar et al., 2014). Therefore, an in-depth study of the tumor microenvironment of GBM could help to reveal its tumorigenic mechanisms. The tumor microenvironment (TME) has attracted more and more attention recently (Yang et al., 2018). TME is composed of a variety of cell types and plays a vital role in tumors (Hanahan and Weinberg, 2000). TME and its function is crucial for understanding the mechanism of tumor development (Duchnowska et al., 2016; Velaei et al., 2016). Estimation of stromal and immune cells in malignant tumor tissues using expression data (ESTIMATE) is an algorithm to help researchers to estimate the proportion of immune cells and stromal cells in tumors based on the gene expression profile (Yoshihara et al., 2013; Li et al., 2016).

Recently, the advances of bioinformatics and high-throughput data have identified potential tumor biomarkers, which could help to develop better prognostic predictions of GBM (Mehta et al., 2010). Weighted gene co-expression network analysis (WGCNA) is a bioinformatics method that could explore the correlation between genes and clinical characteristics and screen crucial genes for further verification (Langfelder and Horvath, 2008; Yuan et al., 2020). In the study, we applied the ESTIMATE algorithm and differential analysis to identify immune-associated genes in GBM for prognosis prediction. We also used the WGCNA to construct a co-expression network and to filter potential gene modules and crucial genes. Our study could provide new opinion to help to find some essential prognostic biomarkers in GBM.

## Materials and Methods

### Description of the Cohort and Sources of Data

The high-throughput RNA-seq data and clinical information of 539 GBM patients were downloaded from the TCGA database. The genes’ expression level of raw count data was quantified as fragments per kilobase million (FPKM) and normalized by log2-based transformation. The samples that lacked overall survival traits were eliminated, and only 412 patients were selected to subsequent analysis. Then, we used the ESTIMATE algorithm to calculate the immune and stromal scores of the samples. A test data contains 237 GBM patients’ expression levels and clinical data was downloaded from the CGGA database (Bao et al., 2014).

### Differential Expression Analysis

We classified the 412 patients into high immune associated and low immune associated groups or high stromal associated and low stromal associated groups by immune score or stromal score based on ESTIMATE analysis. Then, the “limma” R package was used to perform the differential expression analyses between high and low score groups. The DEGs were selected with an absolute log2 fold change ≥0.263 and an adjusted *P*-value <0.05.

### Co-expression Network Construction and Module Identification

The immune associated DEGs were input into the WGCNA to construct co-expression network by WGCNA package. With the help of the function *pickSoftThreshold*, a signed adjacency matrix was calculated to reach approximate scale-free topology of the network (R^2^ >0.8). Then, the weighted adjacency matrix was transformed into a topological overlap matrix (TOM) to minimize effects of spurious associations. A dynamic cut-tree algorithm was used to identify stable modules. Next, the correlation between module eigengene (ME) and clinical data was defined as module significance (MS). The correlation between ME and genes was expressed as module membership (MM). In detail, ME means the first principal component of a given model and represents the gene expression profile of the entire model. MS means the average gene significance of all the genes involved in the module. MM means the correlation between a given gene expression profile and a given model eigengene. Genes with both high gene significance (GS >0.1) and high module membership (MM >0.6) were defined as hub genes.

### Enrichment Analysis and Survival Analysis

KEGG, GO, and Hallmark analysis were performed to explore the potential functions and involved pathways of DEGs. We used the “clusterProfile” R package (Yu et al., 2012) and Metascape web tool (Zhou et al., 2019) to do the analysis.

A Kaplan–Meier curve was used for survival analysis and the curves were used to display the impact on the patients’ survival of candidate genes. Multivariate cox regression analysis was performed to assess whether the genes were independent prognostic factors for patient survival. The “survival” R package was used to perform the above analysis. Furthermore, the OSgbm tool was used to verify the prognostic biomarkers though a combined dataset contains 684 GBM patients (Dong et al., 2019).

## Results

### Identification of Differentially Expressed Genes Related to Tumor Microenvironments

**FIGURE 1:**
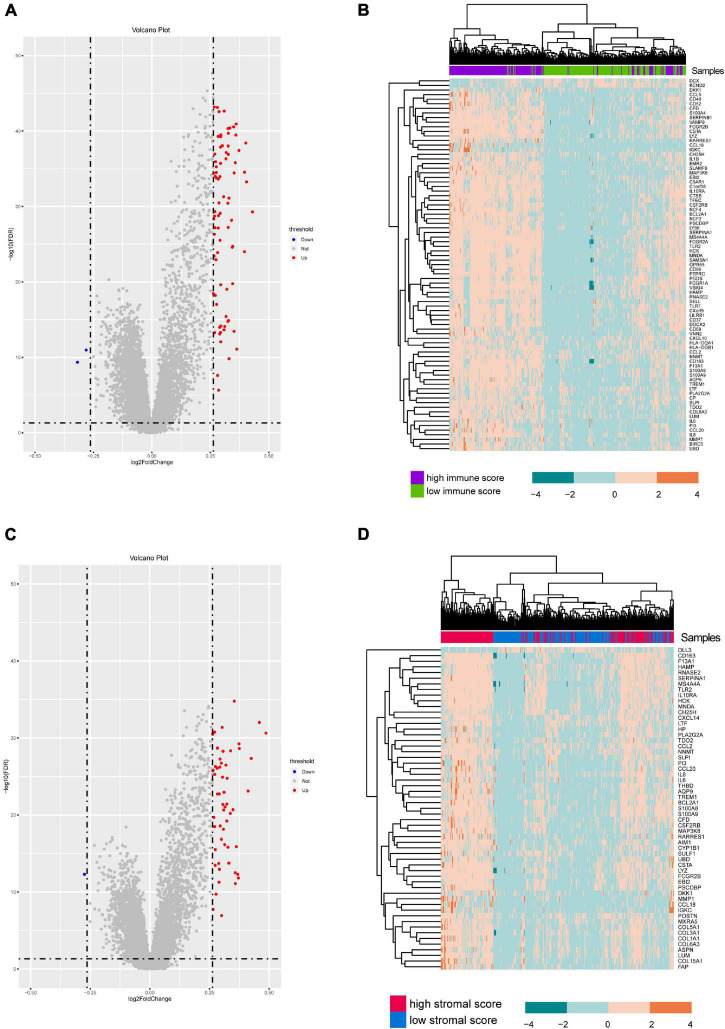
Differential analysis of 412 GBM samples. **(A,C)** Volcano plot shows DEGs between GBM and normal samples. Red represents upregulated DEGs while blue shows the downregulated one (*P* < 0.05). **(B,D)** Heatmap showing the expression level of these differentially expressed genes.

A total of 412 eligible patients’ expression levels and paired clinical data were obtained from the TCGA database. After the ESTIMATE analysis, we distinguished these patients into two groups based on the median value of immune or stromal score. Then, we performed differential expression analysis to identify differentially expressed genes associated with microenvironments. In the immune group, there were 81 DEGs, 79 DEGs were up-regulated, and 2 DEGs were down-regulated (Figures 1A,B). Similarly, 58 genes were differentially expressed according to stromal score, 57 DEGs were up-regulated, and 1 DEG was down-regulated (Figures 1C,D). As shown in Supplementary Figure 2, the immune associated DEGs were mainly enriched in the IL-17 signaling pathway, Toll-like receptor signaling pathway, and phagosome (KEGG pathway) (Supplementary Figure 2A), and humoral immune response, neutrophil activation, and neutrophil mediated immunity (GO terms) (Supplementary Figure 2B). Also, the stromal DEGs were mainly enriched in cytokine-cytokine receptor interaction, chemokine signaling pathway, IL-17 signaling pathway (KEGG pathway) (Supplementary Figure 2C), and acute inflammatory response, leukocyte migration, and response to lipopolysaccharide (GO terms) (Supplementary Figure 2D). Interestingly, the clustering analysis showed that immune-related differential genes could classify GBM patients into two categories, whereas stroma-related differential genes did not have such classification efficacy (Figures 1B,D). In this study, we selected the immune associated DEGs for further analysis.

### WGANA to Obtain the Candidate Genes With Co-expression Pattern

**FIGURE 2:**
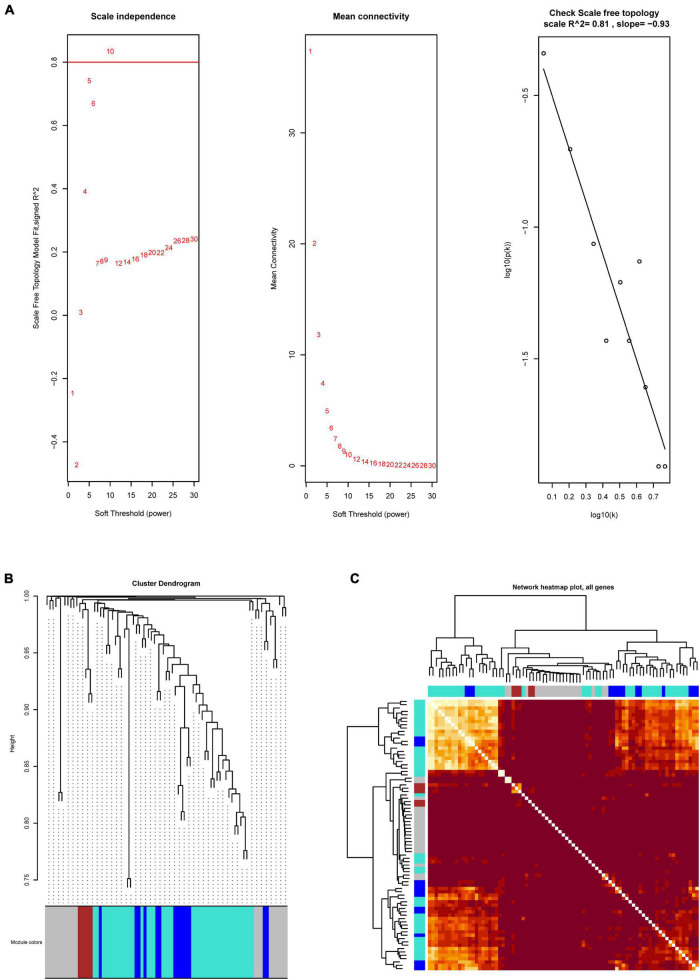
The WGCNA analysis of immune-related DEGs. **(A)** Network topology analysis to select suitable soft-threshold powers. The *x*-axis and *y*-axis reflect the soft-thresholding power and the scale-free topology model fit index, respectively. **(B)** Clustering dendrogram of genes, with dissimilarity based on topological overlap, together with assigned module colors. **(C)** Heatmap showing the expression pattern correlation between these modules.

Based on the differential analyses, the 81 DEGs that are related to the immune system were selected to construct a co-expression network. To prove that the network we constructed is a scale-free network (a network in which a few nodes have many connections, most nodes have a few connections, and the distribution of node degrees in the network conforms to a power-law distribution) and not a random network, we first performed a topology analysis. After a topology analysis of the network, the soft power was set at 10 which the scale independence could reach to 0.81 to perform the subsequent analysis (Figure 2A). Then, we obtained three co-expression modules (MEbrown, MEblue, and MEturquoise) (Figure 2B). It indicated that the immune-related DEGs played three different functions in GBM. Subsequently, we calculated the relationships between the identified modules. It showed that the expression pattern was independent between these modules (Figure 2C).

### A Co-expression Module Was Associated With Targeted Molecular Therapy in Glioblastoma Multiforme

**FIGURE 3:**
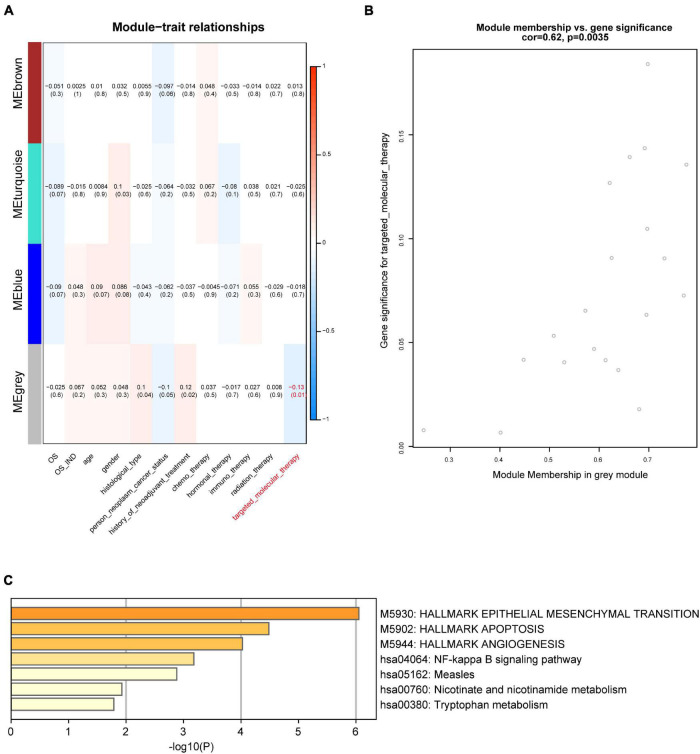
Module-trait associations. **(A)** Module-trait relationships. Each row represents a module when each column indicates a clinical trait. Every cell shows the correlation coefficient and *P*-value. **(B)** Dot plot showing the gray module’s genes significance and module membership in targeted molecular therapy. **(C)** Enrichment analysis of differentially expressed genes in the gray module.

We further explored the three different co-expression modules’ function. We correlated the three modules with GBM patients’ clinical traits to search for potential key modules (Figure 3A). The results illustrated that the gray module was significantly related to targeted molecular therapy in GBM patients. There are 20 candidate genes in this module, 10 out of these genes in the gray module were identified as the hub genes which related to targeted molecular therapy (Figure 3B). We used enrichment analysis to explore the potential function of the hub genes. The result showed that the hub genes were significantly enriched in Apoptosis, NF-kappa B signaling pathway, and tryptophan metabolism. This indicated that the hub genes may regulate GBM progression through these pathways (Figure 3C).

### Three Crucial Hub Genes Associated With Targeted Molecular Therapy Were Potential Prognostic Biomarkers

**FIGURE 4:**
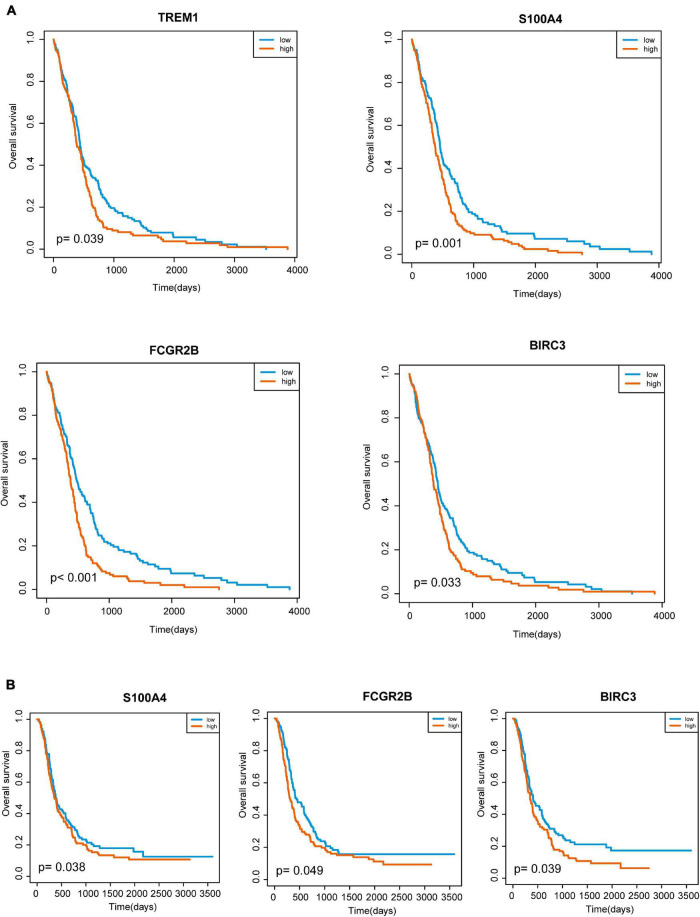
Survival analysis of targeted molecular therapy associated key genes. **(A)** Four genes are potential prognostic biomarkers in TCGA GBM dataset. **(B)** Three out of the four genes are stable survival associated in test data.

**FIGURE 5:**
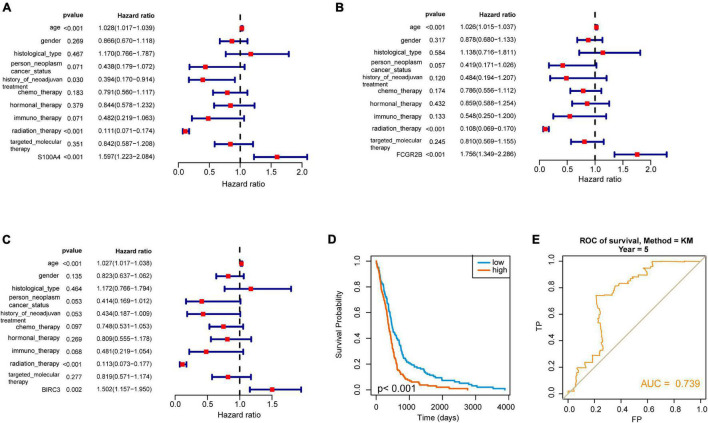
Selection of independent prognostic genes in GBM. **(A–C)** Forest plot showed the hazard ratio of three hub genes (S100A4, FCGR2B, and BIRC3) and suggested that these genes are independent prognostic factors. **(D)** The Kaplan–Meier curve showed that the survival model played an excellent prognostic ability in GBM. **(E)** ROC analysis showed the AUC of the model. It reflected that it is a good prognostic model in GBM.

To further determine these hub genes’ ability and find out the potential prognostic genes, all the 10 targeted molecular therapy associated crucial genes were tested by Kaplan–Meier analysis. The result showed that 4 genes (TREM1, S100A4, FCGR2B, and BIRC3) out of these hub genes were significantly associated with OS in 412 GBM patients (Figure 4A). Then, we selected a dataset that contains 237 GBM samples for the validation. It showed that three crucial genes (S100A4, FCGR2B, and BIRC3) were survival associated (Figure 4B). The multivariate cox regression analysis also found the three genes were independent prognostic factors (Figures 5A–C). A survival model constructed by the three genes also performed a good prognostic efficacy (Figure 5D) and the AUC of the model reached to 0.739 (Figure 5E). Finally, another test set which contained 684 GBM patients’ survival information was also used to testify as to the crucial genes’ prognostic efficacy (Supplementary Figure 2). The result showed that all the three genes are stable prognostic biomarkers and may be the prognostic biomarkers of GBM.

## Discussion

Glioblastoma multiforme is the most malignant brain tumor and requires powerful biomarkers to perform effective treatment (Szopa et al., 2017). High-throughput sequencing provides insights into understanding the pathogenesis and the development of therapeutic biomarkers (Tsimberidou, 2015). Multiple molecular analysis has been used for tumor biology prediction or risk stratification (Chen et al., 2020). To date, the microenvironment has been investigated in numerous cancer studies (Bi et al., 2020; Du et al., 2020; Mao et al., 2020). However, the comprehensive prognostic value of crucial microenvironment-associated biomarkers has not been exploited in GBM.

Here, we applied bioinformatics analysis to integrate high-throughput data from GBM and obtained three microenvironment-associated biomarkers (Supplementary Figure 1). Three potential prognostic biomarkers of GBM were obtained in our study. S100A4 encodes a member of the S100 protein family. This protein family is mainly involved in cell cycle progression and plays a role in microtubule protein polymerization. Aberrant expression of this protein family is associated with tumor metastasis (Sadigh et al., 2019). S100A4 has been reported to be associated with cancer cell migration and metastasis and is important in tumor onset and progression (Atlasi et al., 2016; Liu et al., 2018). In GBM, S100A4 was reported to be associated with the migration and invasion of cancer cells (Zhou et al., 2020). FCGR2B encodes a receptor for the immunoglobulin gamma complex and is involved in the regulation of immune responses and antibody production by B cells (Danzer et al., 2020). FCGR2B has been reported to be associated with anti-GBM disease in Chinese (Zhou et al., 2010). The gene variants of FCGR2B can influence intravenous immunoglobulin response (Shrestha et al., 2011). BIRC3 encodes an IAP family protein. It could inhibit apoptosis by binding to TRAF1 and TRAF2 (Zheng et al., 2010). BIRC3 is a novel prognostic indicator and a potential therapeutic target for cancer (Fu et al., 2019). The expression of BIRC3 could enhance NF-kB translocation and then influence the sensitivity of treatment (Asslaber et al., 2019). Here, we found the three genes played a novel role in the prognosis of GBM. They may be used for further clinical study.

## Conclusion

In this study, we use WGCNA to analyze the high-throughput sequencing data of GBM and identified DEGs associated with the immune microenvironment. Then, the key gene modules associated with GBM patients’ clinical characteristics were obtained. In addition, we identified a gray module consisting of 20 genes which was significantly relevant to the targeted molecular therapy. Ten genes were identified as hub genes and three of them were survival associated. The independent test set of CGGA verified our result. Our results filtered a module and three crucial genes that acted as crucial roles in the prognostic of GBM. The result may provide novel information to improve the prognosis of the tumor.

## Data Availability Statement

The original contributions presented in the study are included in the article/Supplementary Material, further inquiries can be directed to the corresponding author.

## Author Contributions

J-QC and NZ: data collection, data analysis, interpretation, and drafting. Z-HT: study design, study supervision, and final approval of the manuscript. Z-LS, H-GQ, and X-GZ: technical support and critical revision of the manuscript. All authors read and approved the final manuscript.

## Conflict of Interest

The authors declare that the research was conducted in the absence of any commercial or financial relationships that could be construed as a potential conflict of interest.

## Publisher’s Note

All claims expressed in this article are solely those of the authors and do not necessarily represent those of their affiliated organizations, or those of the publisher, the editors and the reviewers. Any product that may be evaluated in this article, or claim that may be made by its manufacturer, is not guaranteed or endorsed by the publisher.
