# Supplementary figures and images for: Integrated Analysis to Obtain Potential Prognostic Signature in Glioblastoma
Source: Front Integr Neurosci. 2022 Jan 5;15:717629. doi: 10.3389/fnint.2021.717629 (PMC8766324; doi:10.3389/fnint.2021.717629)

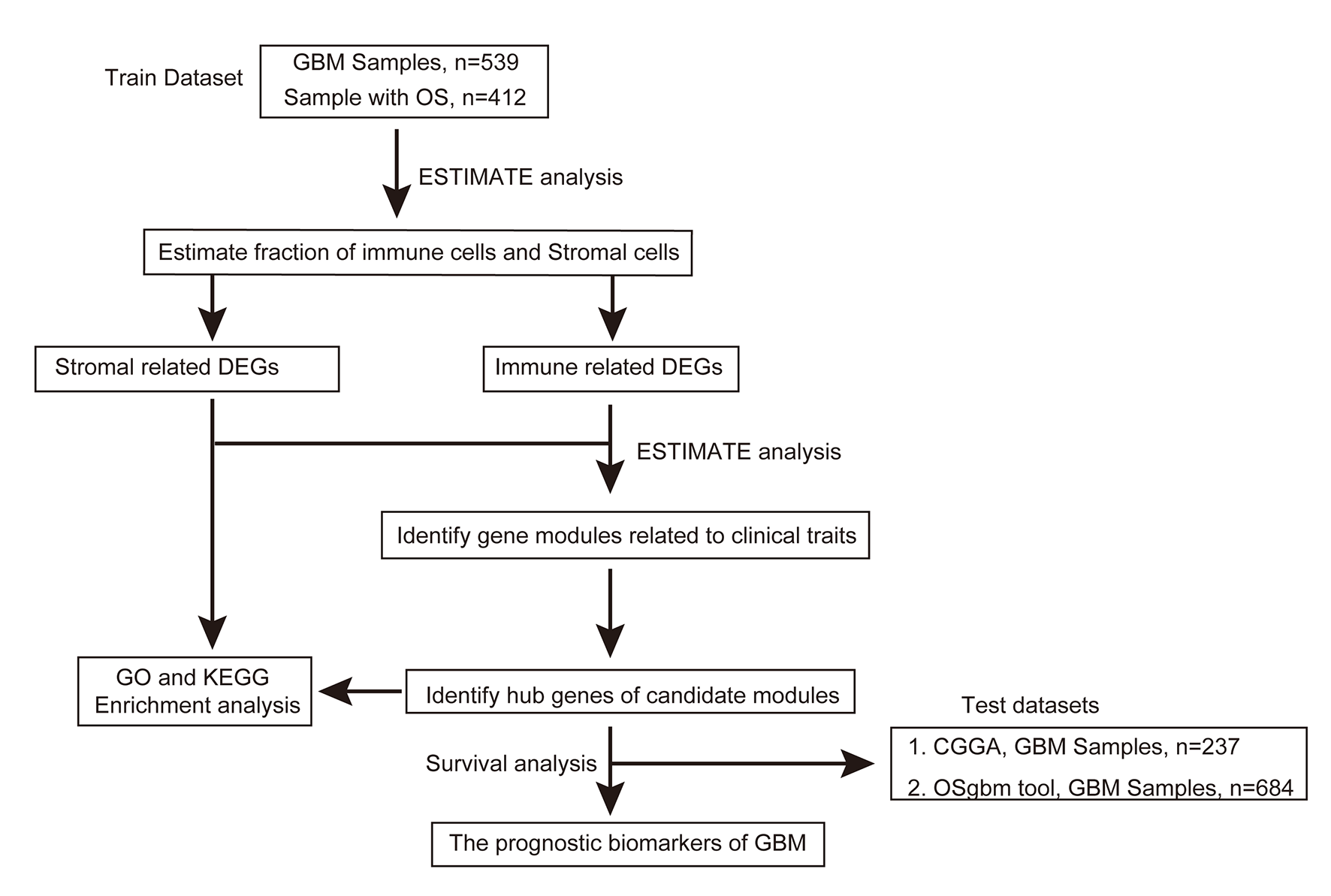

Supplement: Supplementary file 1 [file Image_1.TIF]

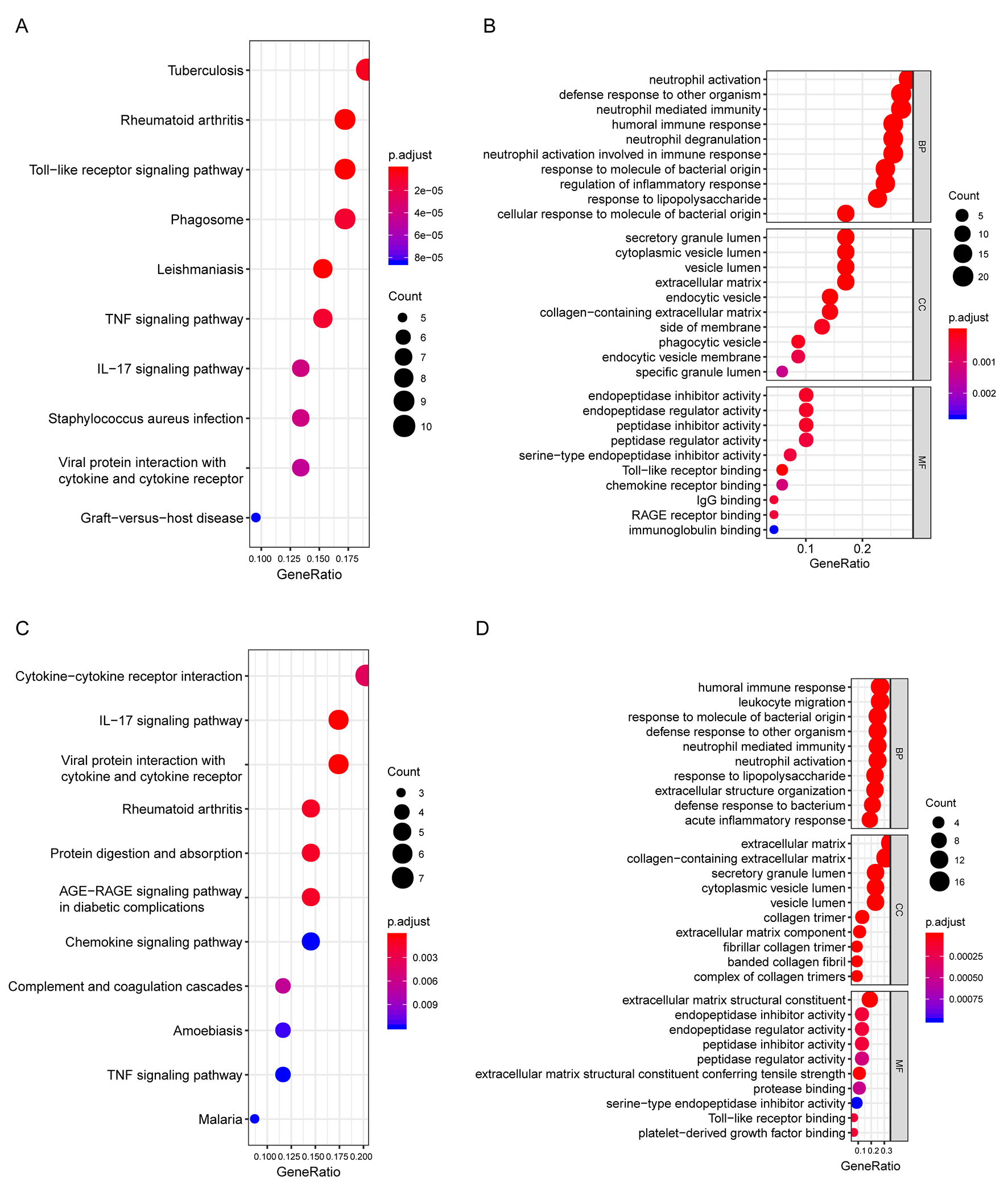

Supplement: Supplementary file 2 [file Image_2.TIF]

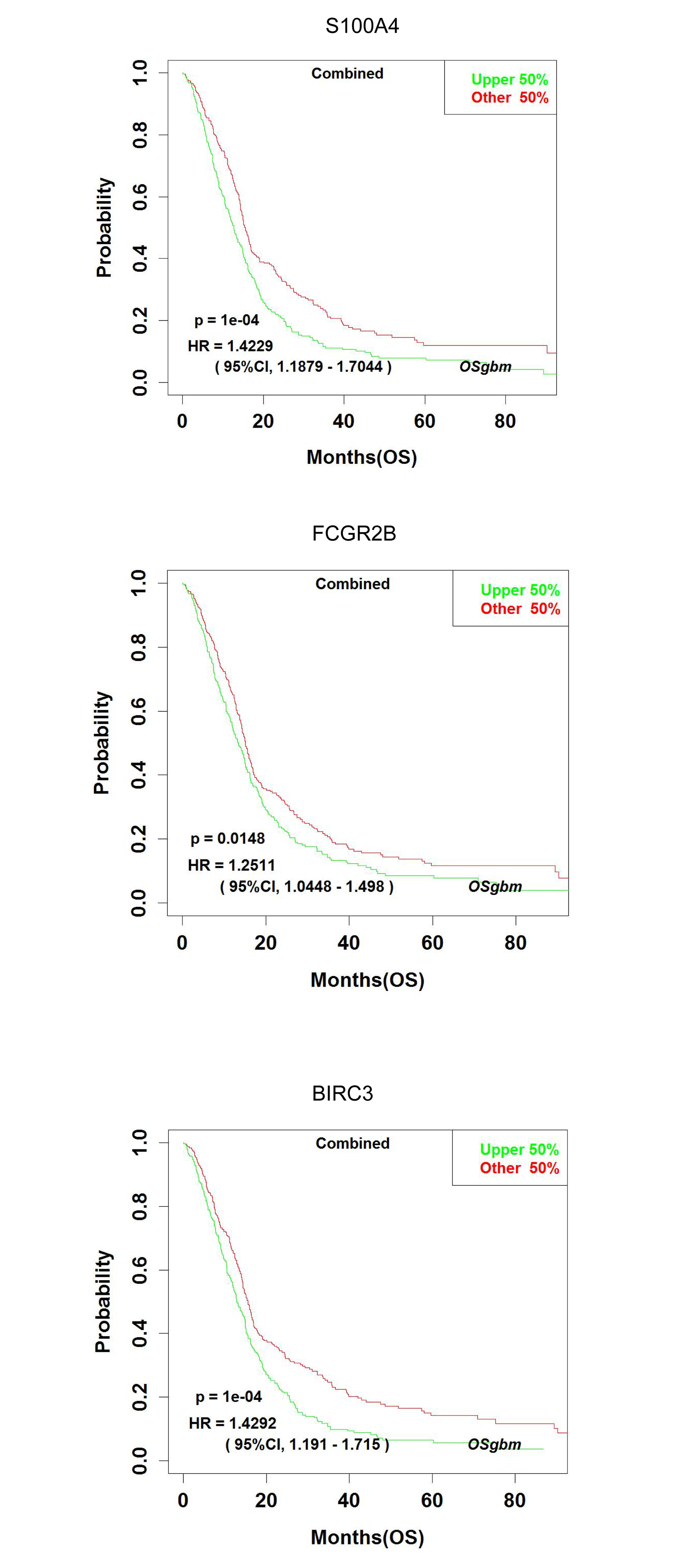

Supplement: Supplementary file 3 [file Image_3.TIF]
